# CGF-HLC-I repaired the bone defect repair of the rabbits mandible through tight junction pathway

**DOI:** 10.3389/fbioe.2022.976499

**Published:** 2022-09-20

**Authors:** Yalin Mao, Miaoling Hu, Li Chen, Xiao Chen, Maohua Liu, Menglian Zhang, Minhai Nie, Xuqian Liu

**Affiliations:** ^1^ Department of Periodontics and Oral Mucosal Diseases, The Affiliated Stomatological Hospital of Southwest Medical University, Luzhou, Sichuan, China; ^2^ Luzhou Key Laboratory of Oral and Maxillofacial Reconstruction and Regeneration, Southwest Medical University, Luzhou, China; ^3^ Department of Stomatology Technology, School of Medical Technology, Sichuan College of Traditional Medcine, Mianyang, China; ^4^ Department of Orthodontics, Mianyang Stomatological Hospital, Mianyang, China

**Keywords:** human-like collagen I, concentrated growth factors, CGF-HLC-I/bio-oss, bio-gide/bio-oss, BMP2, RUNX2

## Abstract

**Background:** The human-like collagen I (HLC-I) combined concentrated growth factors was used to construct CGF-HLC-I composite biomaterials to repair the critical bone defect disease model of rabbit mandible. This study aimed to research the repair mechanism of CGF-HLC-I/Bio-Oss in rabbit mandibular critical bone defect, to provide a new treatment direction for clinical bone defect repair.

**Methods:** The optimal concentration of HLC-I (0.75%) was selected in this study. Nine New Zealand white rabbits were randomly divided into 3 groups, normal control group, Bio-Gide/Bio-Oss and CGF-0.75%HLC-I/Bio-Oss group (*n* = 3, each group). CGF-0.75%HLC-I/Bio-Oss and Bio-Gide/Bio-Oss were implanted into rabbit mandibles, then X-ray, Micro-CT, HE and Masson staining, immunohistochemical staining and biomechanical testing were performed with the bone continuity or maturity at 4, 8 and 12 weeks after surgery. The repair mechanism was studied by bioinformatics experiments.

**Results:** As the material degraded, the rate of new bone formation in the CGF-0.75% HLC-I/Bio-Oss group was better than that the control group by micro-CT. The biomechanical test showed that the compressive strength and elastic modulus of the CGF-0.75%HLC-I/Bio-Oss group were higher than those of the control group. HE and Masson staining showed that the bone continuity or maturity of the CGF-0.75%HLC-I/Bio-Oss group was better than that of the control group. Immunohistochemical staining showed significantly higher bone morphogenetic protein 2 (BMP2) and Runt-related transcription factor 2 (RUNX2) in the CGF-0.75%HLC-I/Bio-Oss group than the control group at 8 and 12 W and the difference gradually decreased with time. There were 131 differentially expressed proteins (DEPs) in the Bio-Gide/Bio-Oss and CGF-0.75%HLC-I/Bio-Oss groups, containing 95 up-regulated proteins and 36 down-regulated proteins. KEGG database enrichment analysis showed actinin alpha 1 (ACTN1) and myosin heavy-Chain 9 (MYH9) are the main potential differential proteins related to osteogenesis, and they are enriched in the TJs pathway.

**Conclusion:** CGF-0.75%HLC-I/Bio-Oss materials are good biomaterials for bone regeneration which have strong osteoinductive activity. CGF-0.75%HLC-I/Bio-Oss materials can promote new bone formation, providing new ideas for the application of bone tissue engineering scaffold materials in oral clinics.

## 1 Introduction

Various biomaterials with different advantages have emerged in biomedical applications. For example, Zhang et al. studied tFNA enables the development of static tFNA-based nanomaterials via engineering of functional oligonucleotides or therapeutic molecules (Zhang et al., 2021). Yang et al. studied A Lysosome-Activated Tetrahedral Nanobox for Encapsulated siRNA Delivery (Gao et al., 2022). Wang et al. found that the tetrahedral framework nucleic acids can alleviate taurocholate-induced severe acute pancreatitis and its subsequent multiorgan injury in mice (Wang et al., 2022). There was some regenerative potential in mandible. However, when the mandibular defects caused by inflammation, infection, tumor, surgery and congenital deformity exceeds a certain size range. The regeneration potential of the mandible will not make the mandibular defect spontaneously healing. Some scholars referred to this type of minimal bone defect that cannot heal spontaneously without secondary intervention as critical size defect (CSD) (Sanders et al., 2014).

The concentrated growth factors (CGF) obtained from autologous venous blood is a cytokine which widely used in clinical practice, and it is the latest generation of platelet concentrate products obtained by centrifugation of autologous blood. The advantages of CGF lie in low cost, low infection rate, good biocompatibility, etc., and it also has osteogenesis induction, and its clinical application is becoming more and more extensive (Akcan and Ünsal, 2020). It has been reported in the literature at home and abroad that CGF contains a large amount of growth factors and fibrin. And it has a strong potential for regeneration and repair, and can induce osteogenic differentiation (Masuki et al., 2016; Li et al., 2021). CGF is mainly used for bone regeneration and has a more significant repair effect on repairing bone defects (Mijiritsky et al., 2021). Meanwhile, CGF membrane can be made into bone defect, combined with scaffold material to guide tissue regeneration (Sahin et al., 2018; Arıcan et al., 2022). Li et al. found that the ability of nano-hydroxyapatite/collagen (nHAC) combined with CGF to promote osteogenesis and repair mandibular defects was stronger than that of nHAC alone (Li et al., 2019). Although CGF was more and more widely used in clinic, there were still some shortcomings such as soft texture, easy collapse of spatial structure, poor plasticity, and unstable fibrin network structure (Wei et al., 2020). Therefore, it needs to be compounded with other biomaterials to improve biomechanical properties. Type I collagen is the main organic component that constitutes the extracellular matrix of bones (Du et al., 2000; Hong et al., 2011; Hempel et al., 2012; Zhou et al., 2020). Due to its excellent biocompatibility, human-like collagen has been used to composite with biomaterials to construct different biomaterial scaffolds, such as bones and blood vessels (Hu et al., 2008). Liao et al. found the hydrogel can enhance the repair of osteochondral defects (Liao et al., 2017).

In this study, CGF was cross-linked with a certain concentration of HLC-I to construct a CGF-HLC-I composite biomaterial, and a classic model of critical bone defect in the mandible of rabbits was established. This study was to explore the feasibility of CGF-HLC-I combined with Bio-Oss in repairing the critical bone defect of the mandible in rabbits and research the proteomic analysis of its promoting mandibular bone tissue aims to mine the differentially expressed proteins and their metabolic pathways that mediate the osteogenesis mechanism. This study revealed how CGF-0.75%HLC-Ⅰ regulated the repair and regeneration of CSD, and provided new ideas for the study of the mechanism of mandible bone defect repair.

## 2 Methods and materials

### 2.1 Animal surgical procedures

The mandibular surgical area was located, and the skin diameter of the incision area with the 11-scalpel was about 15 mm long. The muscles and fascia were separated by blunt incision to expose the bone surface of the mandible. The periosteum was dissected, and drilled 2 mm in front of the mental foramen with a dental slow grinding machine. A classic model of critical bone defect of 10 mm*4 mm*3 mm was constructed from the mandible of rabbits. In the control group, after modeling, no intervention was performed, and the operation area was closed by layered suture. After modeling in the Bio-Gide/Bio-Oss group, Bio-Oss bone meal was implanted into the defect (Normal saline infiltration). Cover the 14 mm*8 mm Bio-Gide periosteum (2 mm covering the edge of the defect), sutured in layers, and closed the operation area. After modeling in the CGF-0.75%HLC-I/Bio-Oss group, 0.025 g Bio-Oss bone meal was implanted into the defect (0.75% HLC-I infiltration). 14 mm*8 mm CGF-0.75%HLC-I composite biofilm was covered (2 mm of defect edge was covered), stratified suture was performed, and the surgical area was closed (Figure 4A).

### 2.2 The optimum concentration of CGF-HLC-I was screened by MTT and Preparation of CGF

The experiment was carried out on 27 6-months female New Zealand (Chongqing Tengxin Biotechnology Co., LTD., License Number: SCXK (Chongqing) 2017–0010) white rabbits weighing 2–2.5 kg. Animals were randomly divided into the control group, the Bio-Gide/Bio-Oss group and the CGF-0.75%HLC-I/Bio-Oss group (*n* = 9 each group). This study has been approved by the Ethics Committee of Biomedical Scientific Research, Affiliated Stomatological Hospital of Southwest Medical University (20180817004). Collection of blood from ear veins of New Zealand white rabbits. CGF fibrin-collagen layers were obtained by centrifugation and then pressed into membranes. CGF-HLC-I composite biomaterials were prepared by compounding with different concentrations of HLC-I (0.00%, 0.10%, 0.25%, 0.50%, 0.75%, 1.00%).

Normal gingival tissue was collected, human gingival fibroblasts were isolated, identified and cultured. The cells were inoculated in 96-well plates (1×10^5^ cells/well) with different concentrations of CGF-HLC-I scaffold in DMEM complete medium, and the blank control group was not inoculated cells. On day 1, 4 and 7, DMEM complete medium was replaced with low-sugar DMEM. Then, the cells were incubated at 37°C, 5% CO_2_ for 12 h, added with MTT solution (10 μl/well) and incubated for 4 h in dark. Finally, the absorbance of each well was measured at 490 nm on an ELISA reader.

### 2.3 Water absorption test of CGF membrane and CGF-0.75%/HLC-I composite biomaterials

The prepared CGF membrane and CGF-0.75% HLC-I composite biofilm were cut into 12 mm × 8 mm rectangular membrane. The CGF-0.75% HLC-I composite biofilm was set as group A (*n* = 3), numbered A1, A2, A3, and CGF membrane as group B (*n* = 3) were numbered as B1, B2, B3. After drying in a freeze dryer for 24 h, the membranes were taken out and weighed as W_0_. Then the dried membranes were immersed in normal saline and placed in a refrigerator at 4°C. 24 h later, the normal saline was wiped off the surface of the biofilm with filter paper, then weighed and marked as W_1_, Water absorption W = (W_1_-W_0_)/W_0_ × 100%.

### 2.4 Biomechanical properties of CGF membrane and CGF-0.75% HLC-I composite biomaterials

Six samples of CGF membrane and CGF-0.75%HLC-I composite biofilm were prepared and immersed in physiological saline respectively to preserve moisture. The maximum capacity of 100 N sensor was installed on INSTRON 5965 electronic universal tester (United States, The United States strong Group), and the stretching fixture was installed. The working parameters of the instrument were adjusted and set. The initial stretch length of CGF membrane and CGF-0.75%HLC-I composite biofilm were maintained at 15 and 8 mm, respectively. The tensile test was carried out at a rate of 10 mm/min until the membrane structure broke. In this process, the trend of the curve and the abscissa and ordinate values on the curve were observed and recorded. The stress-strain curve and stress-time curve were drawn.

### 2.5 Ultrastructure of CGF and CGF-0.75% HLC-I composite biomaterials

The fresh CGF membrane and CGF-0.75% HLC-I composite biofilm samples were prepared. After 12 h, the samples were taken out, rinsed with PBS buffer, and then dehydrated. The samples were quickly cured by placing them in a 4°C refrigerator. Finally, the samples were placed in a coating machine to dry the samples and sprayed with metal films, and the ultrastructure of the samples was observed by scanning electron microscopy.

### 2.6 *In vitro* cytotoxicity evaluation of CGF-0.75% HLC-I composite biomaterials

The cells in the CGF-0.75% HLC-I group and the CGF groups were laid at the bottom of the 96-well plate. 100 μl/well cell suspension was added to 96 wells and cultured in 5% CO_2_, 37°C for 24, 48 and 72 h, respectively. 10 ul CCK-8 solution was added to each well and incubated for 3 h in the cell culture box. OD value was measured at 450 nm by Elisa (mean value was taken for 3 times), and recorded. The relative growth rate (RGR) was calculated to evaluate the cytotoxicity grade as the following formula: RGR= (experimental group OD value-blank group OD value)/(control group OD value - blank group OD value)×100%.

### 2.7 Establishment of animal models

Before animal experiment, abstain from food and water for 8 h, weigh and record. Anesthesia was slowly injected into the ear margin vein. After anesthesia, the hair of the right mandible area was removed and disinfected. 2 ml 0.5% lidocaine was used for local infiltration and anesthesia in the operation area, followed by routine towel spreading. The mandibular surgical area was located. The skin diameter of the surgical area was about 15 mm. The muscle and fascia were cut and bluntly separated to expose the bone surface of the mandible. The mental nerve was separated and protected, the periosteum was cut open, and the periosteum was peeled off. The periosteum was separated 2 mm in front of the mental foramen with a dental slow grinding machine and split drill. A classical critical bone defect model of 10 mm × 4 mm×3 mm was constructed with the mandibles of each group of rabbits (During the defect construction process, sterile saline was continuously rinsed to cool down the operation area to avoid osteonecrosis caused by high local temperature.) Bio-Gide/Bio-Oss group was implanted with Bio-Oss bone meal (saline infiltration) after modeling. The 14 mm × 8 mm Bio-Gide periosteum was covered (covering the defect edge by 2 mm), and the surgical area was closed by stratified suturing.

### 2.8 Histological evaluation

24, 48 and 72 h after surgery, the CGF membrane and CGF-0.75% HLC-I complex biofilm samples were placed in 4% paraformaldehyde solution, fixed for 48 h and labelled in groups. The samples were dehydrated, decalcified, embedded and cut into continuous slices with a thickness of 5 µm. HE staining and masson staining were carried out by conventional methods. Finally, the tablets were sealed with neutral resin and preserved by forward microscopy. Bone morphogenetic protein 2 (BMP2) and runt-related transcription factor (RUNX2) were detected by SP-2300 immunohistochemical staining kit. The samples were decalcified and then embedded in paraffin wax. Subsequently, the samples were cut along the sagittal plane and then dewaxed. The resulting sections were then incubated with primary antibodies (Anti-BMP2 antibody, Beijing Boaosen Biotechnology Co. LTD., China) after antigens were recovered using 0.05% parenzyme for 20 min and blocked by BSA. Next, the secondary antibody Primary antibody myosin heavy-Chain 9 (MYH9), RAB8A, actinin alpha 1 (ACTN1), RAB13, MYL2, AMPK alpha-1, phosph-AMPK alpha-1, RUNX2, BMP2, beta Actin (Goat Anti-Mouse (ab205719, Abcam, United Kingdom) and 3, 30-diaminobenzidine (DAB, ab64261, Abcam, United Kingdom)) was used for secondary staining. The images were acquired using a 1 × 71 PH inverted microscope (OLYMPUS, Japan) acquisition system. For the immunohistochemical analysis, BMP2 and RUNX2 positive expression area were shown in brown.

### 2.9 Western blot

To obtain whole protein cell extracts for Western-blot analysis, cells were scraped in the following buffer: 20 mM Tris–HCl (pH 8.0), 420 mM NaCl, 2 mM EDTA, 2 mM Na3VO4, and 1% (v/v) Nonidet P-40, supplemented with a cocktail of protease inhibitors. Cells were then passed several times through a 20-gauge syringe and centrifuged at 16,000× *g* for 20 min at 4°C. Proteins in homogenate were determined using the Bio-Rad protein assay kit. Lyophilized bovine serum albumin (BSA) was used as a standard. Total cell proteins were dissolved in sodium dodecyl sulphate (SDS) sample buffer and separated on 10% (w/v) SDS gels. Separated proteins were then transferred electrophoretically onto a nitrocellulose membrane (Pall, East Hills, NY, United States). Equal protein loading was confirmed by Ponceau S staining. The filter was blocked with 5% (w/v) non-fat dried milk in buffered saline.

Blots were incubated with specific primary antibodies and the immune complexes were detected using appropriate peroxidase-conjugated secondary antibodies and enhanced chemiluminescent detection reagent (Amersham International, Little Chalfont, United Kingdom) (Rochira et al., 2013). Densitometric analysis was carried out on the Western-blots by using the ChemiDoc MP Image System (BioRad, Hercules, CA, United States).

### 2.10 MicroCT

The samples of control group after 12 weeks, Bio-Gide/Bio-Oss groups and CGF-0.75%HLC-I/Bio-Oss were scanned by MicroCT. The voltage was set to 100 kV and the electric current to 500 uA, and the scanned images were analyzed by Inveon Research Workplace software. With the constructed critical bone defect area as Region of interest (ROI), the bone cell analysis parameters of critical bone defect area of rabbit mandible were obtained. The regeneration ability of bone tissue repair and healing was evaluated from MicroCT.

### 2.11 Proteomic analysis of new bone tissue

#### 2.11.1 Construction of critical bone defect model

Nine New Zealand white rabbits were randomized into 3 groups (A: control group, B: Bio-Gide/Bio-Oss, C: CGF-0.75%HLC-Ⅰ/Bio-Oss), with 3 rabbits in each group. The experimental animals in each group were fasted for 12 h before operation, fixed on a constant temperature rabbit platform, and placed in a supine position. 3% pentobarbital sodium per 30 mg/kg injection. A needle was inserted into the right mandibular mental foramen corresponding to the body surface, and 1 ml of lidocaine was injected for local anesthesia. 2 mm anterior to the mental foramen, along the upper edge of the mandible, a 10 mm × 4 mm × 3 mm bone defect was prepared with a slow-machine split drill. After the bone defect was completed, the surgical area was cleaned, rinsed, and disinfected to repair the bone defect: Group A: After flushing with normal saline, the bone defect was directly sutured in layers. Group B: After repeated flushing of the bone defect with normal saline, 0.5 mg Bio-Oss bone filling material was implanted. The Bio-Gide biofilm with a size of 14 mm × 8 mm was soaked with normal saline to cover at least 2 mm outside the edge of the bone defect, and the Bio-Gide biofilm was fixed. Group C: After flushing the bone defect with normal saline, implant 0.5 mg Bio-Oss bone filling material (soaked with 0.75% HLC-I solution). Cover and fix the HLC-I/CGF membrane prepared before operation, cover at least 2 mm outside the edge of the bone defect, and suture in layers. Normal feeding and water feeding were given after operation, and amoxicillin and feed were given for 7 days. The experimental animals were euthanized 3 months after the operation. Immediately after execution, the right mandible was isolated under aseptic conditions as possible, and cut off from the healthy bones at both ends (including healthy bones and 15 new bones). After being thoroughly rinsed with normal saline, each new bone was divided into 3 parts and stored in a −80°C refrigerator.

#### 2.11.2 Library identification and protein quantification

Raw data files were searched using Proteome Discoverer software (PD) (version 2.4.0.305, Thermo Fisher Scientific) and the built-in Sequest HT search engine. Searched using the Rabbit (Uniprot-Oryctolagus cuniculus) protein database. The proteins with significant differences in protein quantification between the CGF-0.75%HLC-I/Bio-Oss and Bio-Gide/Bio-Oss groups were regarded as valuable differentially expressed proteins. Statistical basis: Student’s t-test or chi-square *p* < 0.05 and FOLD CHANGE (FC) > 1.2 or FC < 0.83. The COG orthologous protein database and the KOG database were used to analyze the homologous protein clusters of histone C proteins, and the KEGG database was used to search and analyze the related signaling pathways that DEPs were most involved in regulation.

### 2.12 Statistical analysis

All the experiments were independently repeated more than three times. The data of each group are expressed as the mean ± SD. SPSS 22.0 software was used for data processing. Variance and chi-square test were used to analyze the data. When *p* < 0.01 for the test parameters and *p* < 0.05 for the nonparametric tests were observed, the differences were considered to be statistically significant.

## 3 Results

### 3.1 The optimum concentration of CGF combined with HLC-I

**FIGURE 1:**
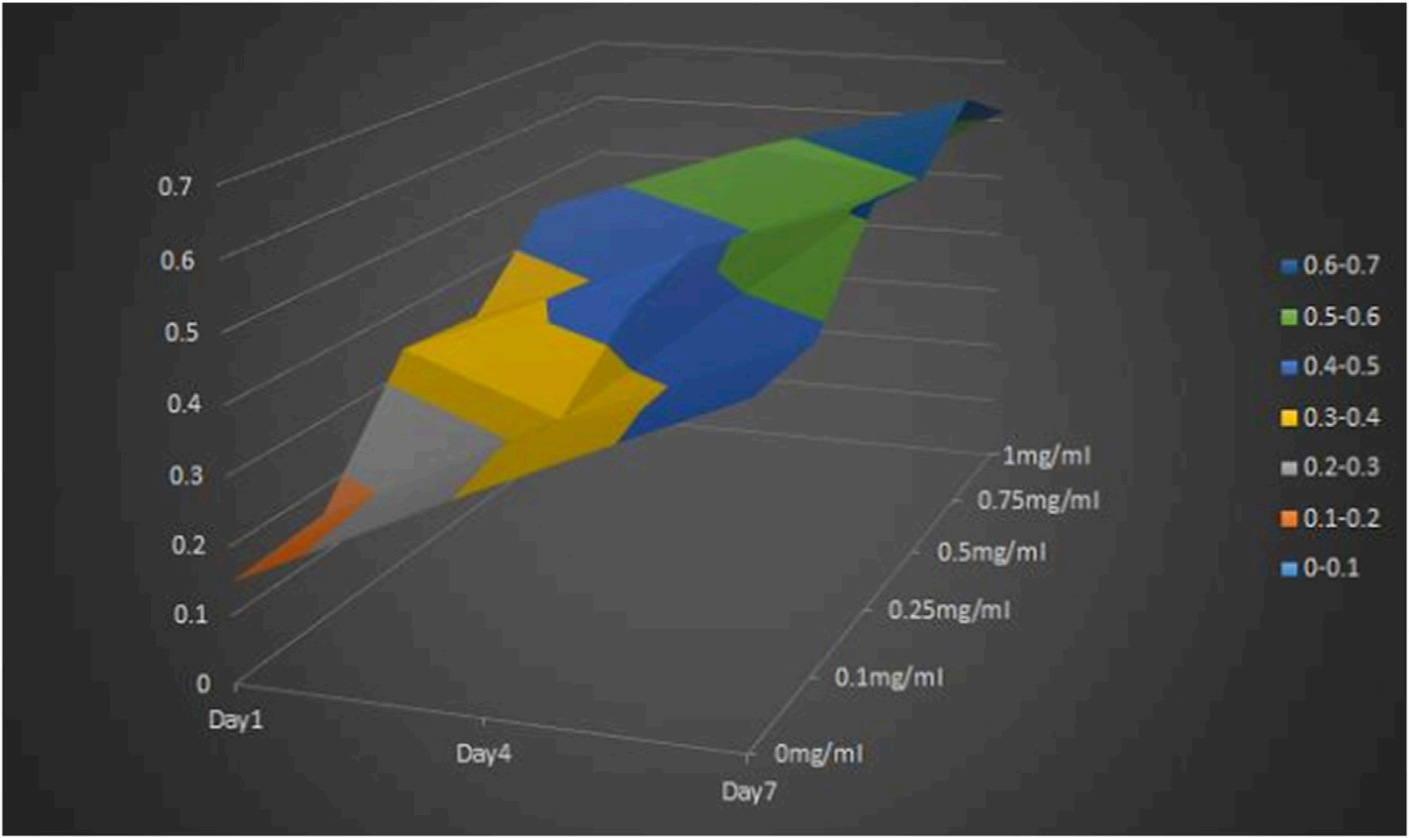
MTT method for screening the optimal concentration of the cross-linking compound of HLC-I and CGF.

MTT results showed that the OD value of cells cultured on CGF-HLC-I composite biomaterials with different concentrations increased with time. However, the cell proliferation of CGF-0.75% HLC-I composite biomaterial was higher than that of other concentrations at 1, 4 days and 7 days. It showed that the combination of 0.75% HLC-I and CGF was more conducive to cell proliferation, that is, the optimal concentration of cross-linking between HLC-I and CGF was 0.75% (Figure 1).

### 3.2 HE staining and masson staining results of CGF membrane and CGF-0.75% HLC-I composite biofilm

**FIGURE 2:**
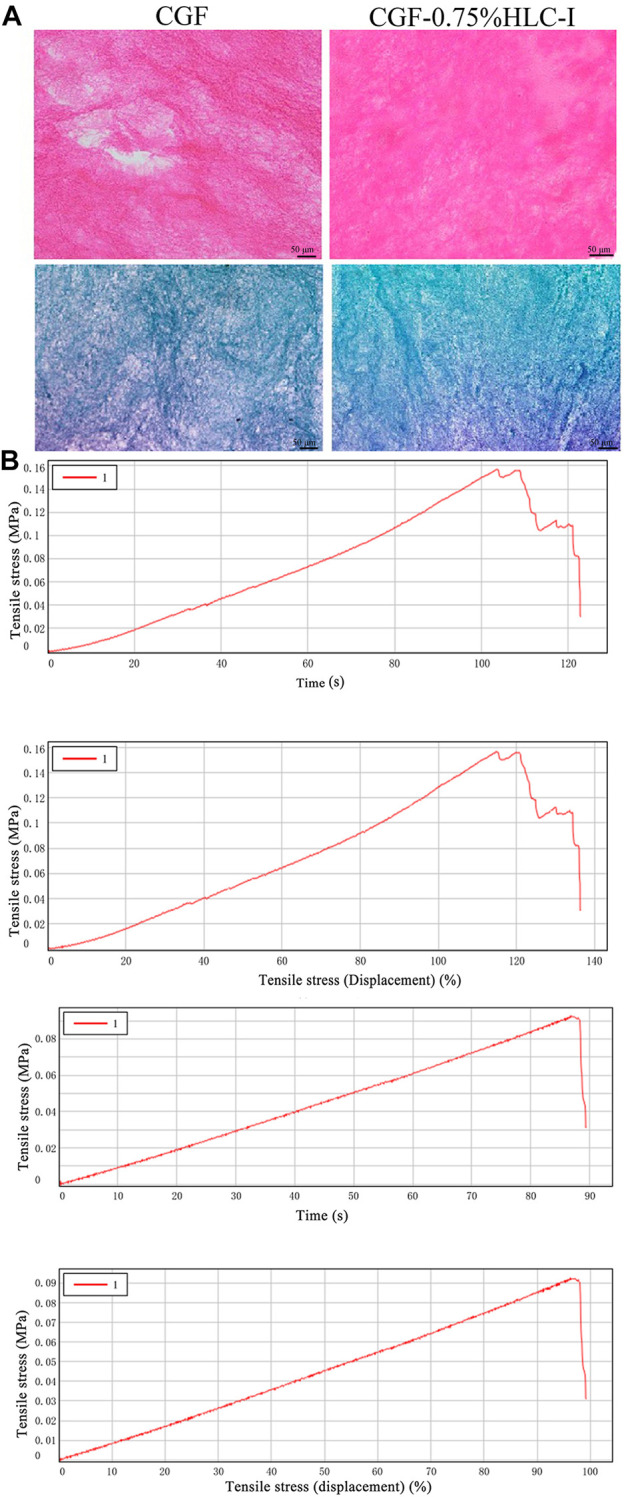
**(A)** HE staining of CGF and CGF-0.75%HLC-I composite biomaterials (HE, ×400); Masson staining of CGF and CGF-0.75%HLC-I composite biomaterials (Masson, ×400); **(B)** CGF, CGF-0.75%HLC-I stress-time diagram and stress-strain diagram.

CGF membrane showed a large number of red stained fibrin interwoven in a network structure. The fibrin net was relatively loose and had a slightly larger porosity. CGF -0.75% HLC-I composite biofilm fibrin mesh arrangement was more dense and detailed than CGF membrane, and the porosity was small (Figure 2A). The arrangement of collagen fibers in CGF membrane was relatively loose, while the arrangement of collagen fibers in CGF-0.75% HLC-I composite biofilm was more dense (Figure 2A).

### 3.3 Water absorption test results of CGF membrane and CGF −0.75% HLC-I composite biofilm

The water absorption of CGF film was (346.00 ± 2.00) %. The water absorption of CGF-0.75%HLC-I film was 344.00 ± 1.00%. The results showed that there was no significant difference in water absorption between CGF membrane and CGF-0.75% HLC-I composite biofilm (*p* > 0.05, Table 1).

**TABLE 1:** Comparison of hydroscopicity test between CGF membrane and CGF-0.75% HLC-I composite biofilm (‾X ± S).

Support Material	*n*	Water Absorption (%)
CGF	3	346.00 ± 2.00
CGF-0.75%HLC-I	3	344.00 ± 1.00

### 3.4 Biomechanical properties of CGF membrane and CGF-0.75% HLC-I composite biofilm

The peak tensile stress of CGF-0.75% HLC-I composite biofilm and CGF membrane were 0.14 ± 0.01 MPa and 0.10 ± 0.01 MPa, respectively. The tensile force of CGF-0.75% HLC-I composite biofilm reached the peak at 104.63 ± 14.33 s, while that of CGF membrane reached the peak at 85.64 ± 8.23 s. The elastic modulus of CGF-0.75% HLC-I composite biofilm was 0.17 ± 0.04 MPa, which was slightly larger than that of CGF membrane 0.12 ± 0.01 MPa. The tensile displacement, time and displacement of CGF-0.75% HLC-I composite biofilm were greater than those of CGF membrane, and the difference was statistically significant (*p* < 0.05, Table 2). Stress-time curves showed that CGF-0.75% HLC-I composite biofilms reach stress peak later than CGF films. The stress-strain curve showed that the tensile stress decreased slowly after the CGF-0.75% HLC-I film reached its peak (Figure 2B).

**TABLE 2:** Biomechanical properties of CGF membrane and CGF-0.75%HLC-I composite biofilm (X ± S).

Material	Maximum Value (N)	Tensile Displacement at Maximum Value (mm)	Tensile Stress at Maximum Value (MPa)	Time at Maximum Value (s)	Elastic modulus at Maximum Value (MPa)
CGF	0.62 ± 0.07	14.25 ± 1.37	0.10 ± 0.01	85.64 ± 8.23	0.12 ± 0.01
CGF-0.75%HLC-I	0.93 ± 0.06^*^	17.42 ± 2.39^*^	0.14 ± 0.01^*^	104.63 ± 14.33^*^	0.17 ± 0.04^*^

Material	Tensile Displacement at Break (mm)	Tensile Stress at Break (MPa)	Time at Break (s)	Displacement at Break (mm)
CGF	14.50 ± 1.44	0.06 ± 0.03	87.09 ± 8.65	14.50 ± 1.44
CGF-0.75%HLC-I	28.56 ± 8.70^*^	0.05 ± 0.03	171.45 ± 52.18^*^	28.56 ± 8.70^*^

Compared with CGF, ^*^ *p* < 0.05.

### 3.5 Ultrastructural results of CGF membrane and CGF-0.75% HLC-I composite biomaterial

**FIGURE 3:**
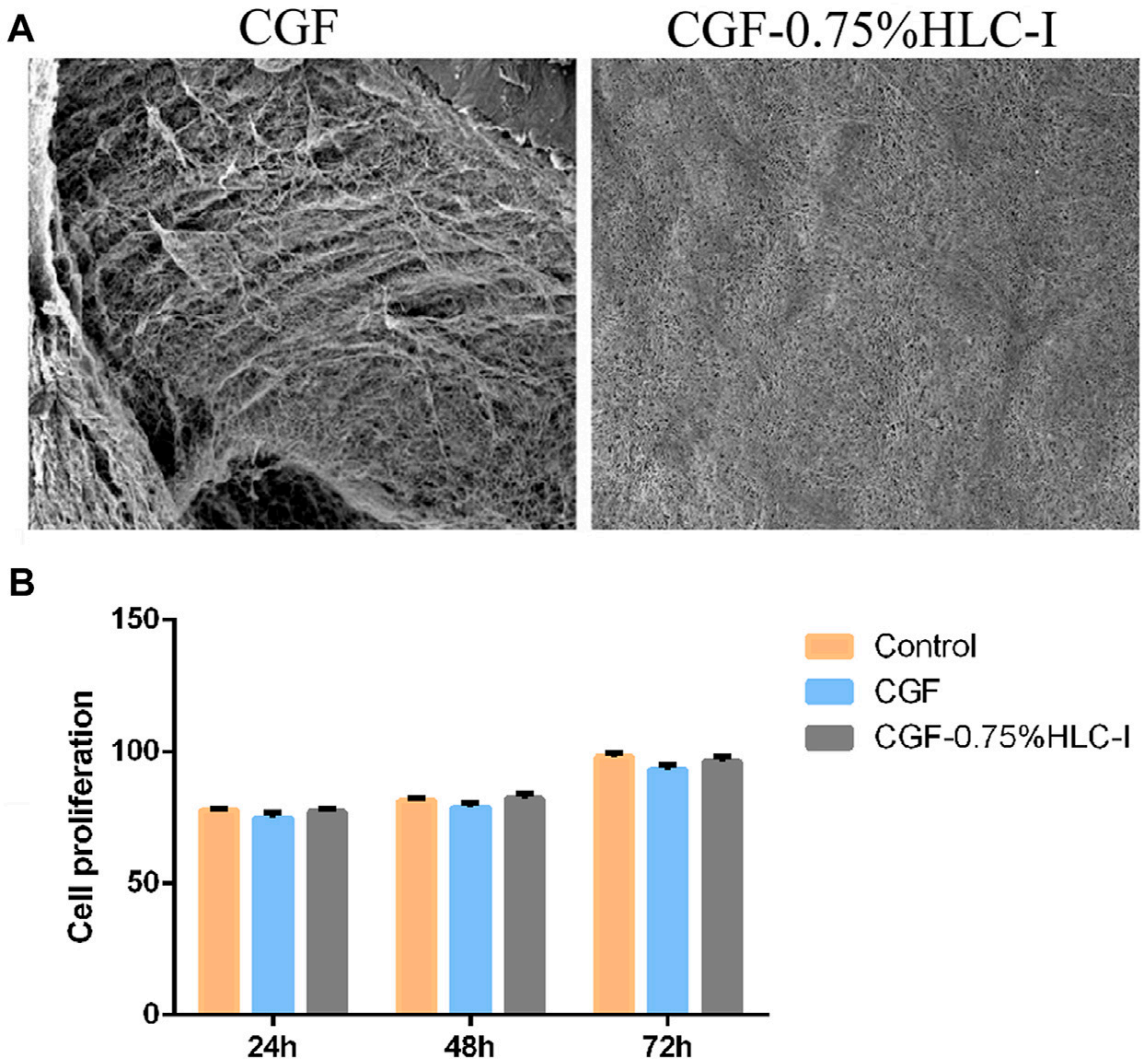
**(A,B)** The ultrastructure of CGF **(A)** and CGF-0.75%HLC-I composite biomaterials **(B)** (SEM, ×800). **(B)** The cytotoxicity of CGF and CGF-0.75% HLC-I composite biomaterials was determined by CCK-8.

The SEM results showed that the fibrin network structure of the CGF-0.75%HLC-I composite biofilm was uniform and fine, and the fibrin network structure was filled by HLC-I, which reduced the porosity. In the CGF membrane, a large amount of fibrin aggregates into a loose and porous three-dimensional network structure (Figure 3A).

### 3.6 *In vitro* cytotoxicity evaluation results of CGF-0.75%HLC-I composite biomaterials

The results of CCK-8 *in vitro* cytotoxicity test showed that the relative cell proliferation rate of CGF-0.75% HLC-I composite biofilm group and CGF film group at 24, 48, 72 h was not statistically significant (*p* < 0.05), and at each time point the relative cell proliferation rate was ≥75%. The relative cell proliferation rate of the CGF-0.75%HLC-I composite biofilm group and the CGF film group at 24, 48, and 72 h was significantly different (*p* < 0.05), and cell proliferation showed a gradual upward trend with time (Figure 3B).

### 3.7 The results of bone mineral density

**FIGURE 4:**
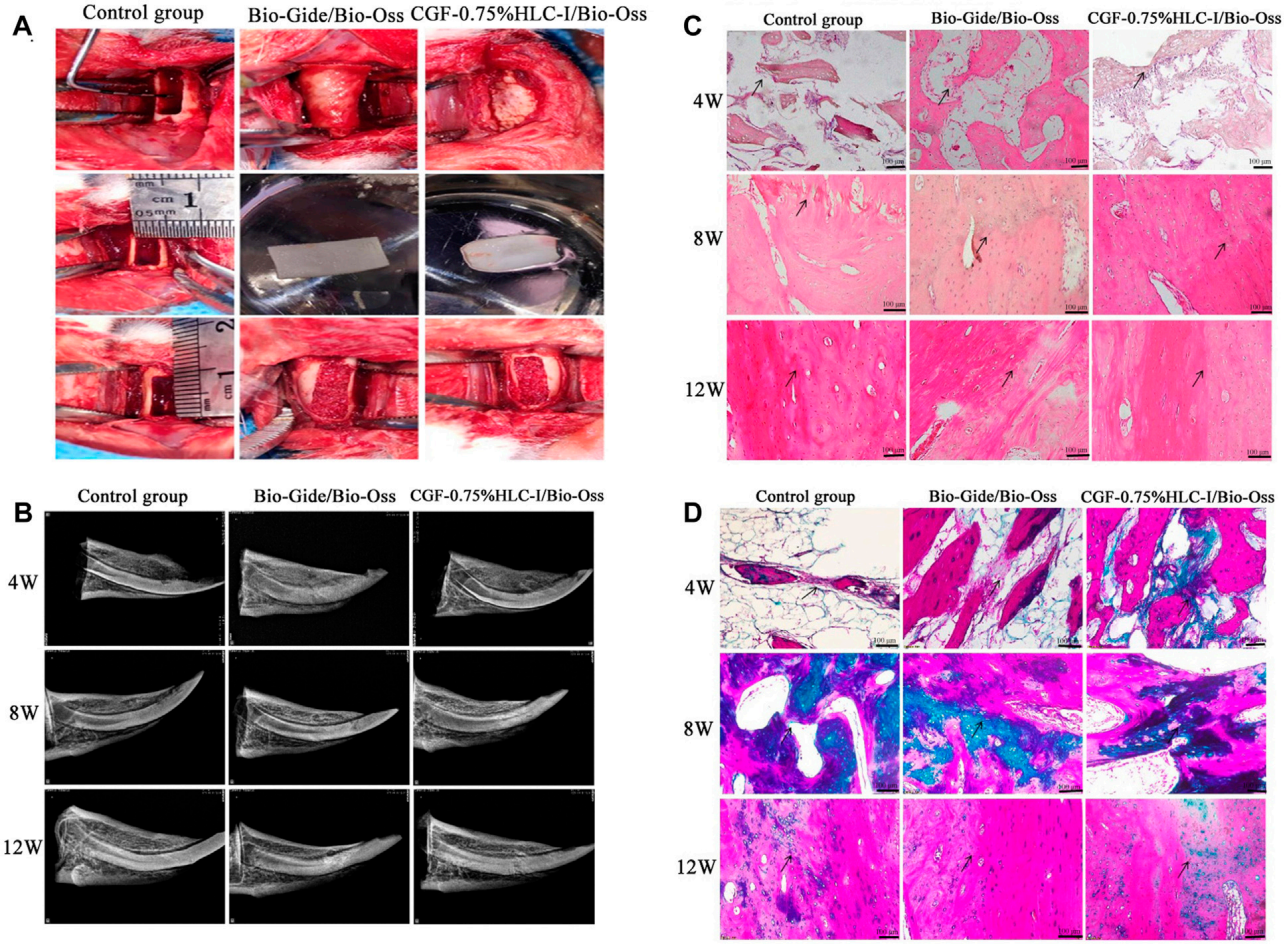
**(A)**. Construction of critical-size bone defect; **(B)** X-ray images of control group, Bio-Gide/Bio-Oss group and CGF-0.75%HLC-I/Bio-Oss group; **(C)** Histological observation of control group, Bio-Gide/Bio-Oss group, and CGF-0.75% HLC-I/Bio-Oss group (HE, ×200); **(D)** Histological observation of control group, Bio-Gide/Bio-Oss group, and CGF-0.75% HLC-I/Bio-Oss group (Masson, ×200).

At 4, 8, and 12 W after operation, the bone mineral density of the mandibular defect in the Bio-Gide/Bio-Oss group and the CGF-0.75%HLC-I/Bio-Oss group gradually increased. At 12 w, there was no obvious boundary between bone mineral density and surrounding bone tissue. In the control group, the low-density area in the center of the mandibular defect area gradually decreased, and a small amount of bone density in the center of the defect area was slightly lower than that of the surrounding bone tissue (Figure 4B).

### 3.8 HE staining results of bone formation

At 4, 8, and 12 W after operation, new fibrous bone tissue, scattered osteoclasts and a few osteoblasts were seen in the Bio-Gide/Bio-Oss group and the CGF-0.75%HLC-I/Bio-Oss group. At 8 W, a large number of new mature bone formation and obvious new bone fracture line can be seen. Compared with the control group at 12 W, more dense and mature bone trabeculae were regularly arranged. In the control group at 12 W, a small amount of osteoclasts existed in the fibrous connective tissue and the trabecular bone was thick and regularly arranged (Figure 4C).

### 3.9 Masson staining results of collagen fibers

4 W after operation, the number of fibroblasts and collagen fibers in the Bio-Gide/Bio-Oss group and CGF-0.75%HLC-I/Bio-Oss group gradually increased. 8 W after operation, the arrangement of fibroblasts was gradually regular, and the number of collagen fibers stained green was more than that of the control group. The arrangement of collagen fibers in the control group at 12 W after operation tended to be regular (Figure 4D).

### 3.10 Immunohistochemical analysis of BMP2 and RUNX2 protein expression in new bone tissue

4 W after operation, the Bio-Gide/Bio-Oss group and the CGF-0.75%HLC-I/Bio-Oss group showed the formation of new fibrous bone, a few osteoblasts and scattered osteoclasts. BMP2 and RUNX2 were positively expressed in the newly formed cartilage matrix, osteoblasts and granulation tissue stroma. In the control group, there was no new cartilage matrix formation, a small amount of fibrous connective tissue, and more osteoclasts. BMP2 and RUNX2 were positively expressed in the granulation tissue stroma.

8 W after operation, a large number of new mature bone and osteoblasts were seen in the Bio-Gide/Bio-Oss group and CGF-0.75%HLC-I/Bio-Oss group, and osteoclasts were scattered. Uniform positive expression of BMP2 and RUNX2 in new mature bone, osteoblast and granulation tissue. In the control group, a small amount of new bone was formed, and osteoblasts, inflammatory cells and osteoclasts existed at the same time. Cartilage matrix, osteoblasts, granulation tissue interstitial BMP2 and RUNX2 were positively expressed.

**FIGURE 5:**
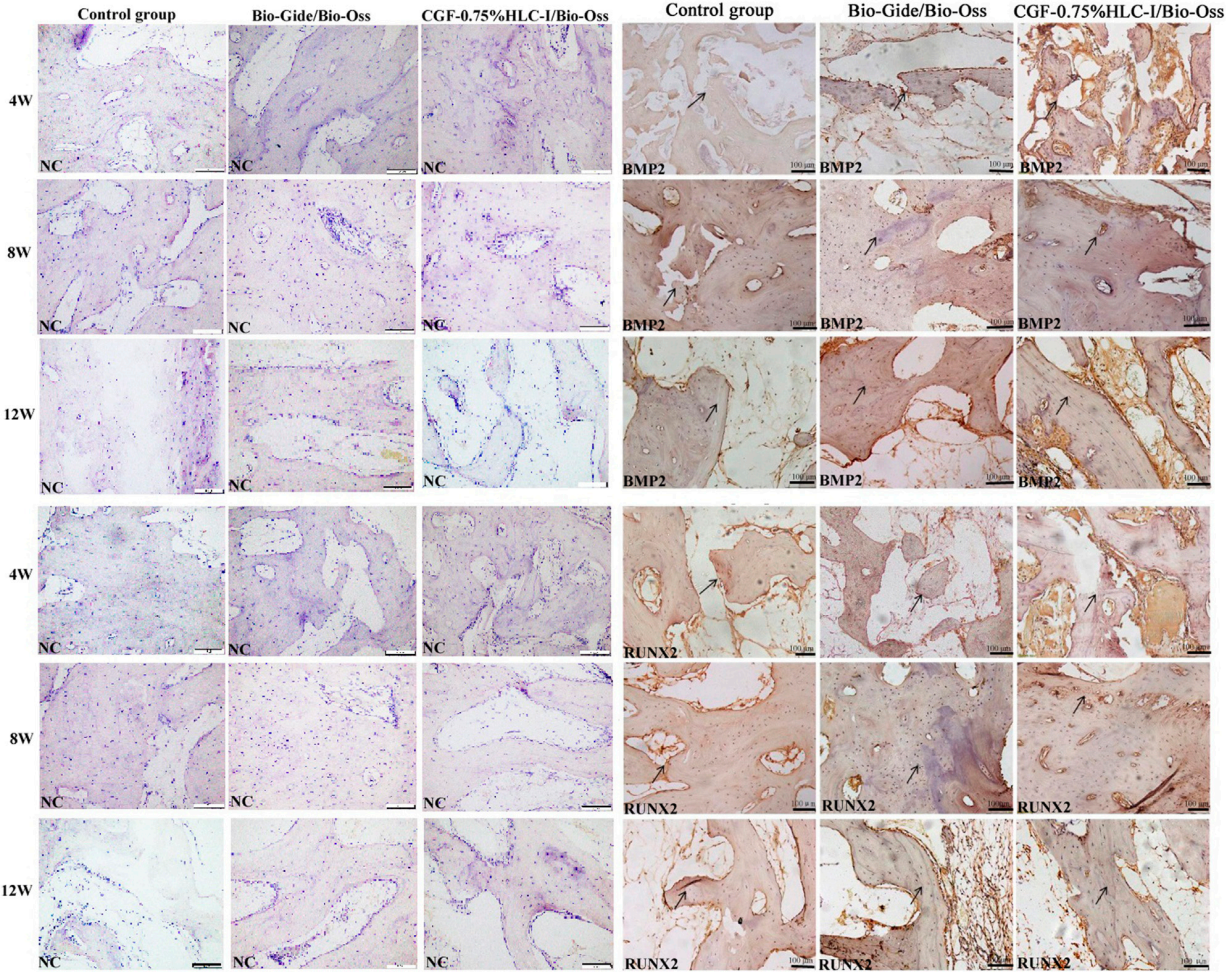
Immunohistochemical analysis of BMP-2 protein expression in control group, Bio-Gide/Bio-Oss group, CGF-0.75%HLC-I/Bio-Oss group (SP, ×200); Immunohistochemical analysis of RUNX-2 protein expression in control group, Bio-Gide/Bio-Oss group, CGF-0.75%HLC-I/Bio-Oss group (SP, ×200). NC- Negative Control.

At 12 W after operation, in the Bio-Gide/Bio-Oss group and the CGF-0.75%HLC-I/Bio-Oss group, the thickness of the trabecular bone was densely and regularly arranged, the fracture line of the new bone tissue was seen, and osteoblasts were scattered. BMP2 and RUNX2 were positively expressed. In the control group, the trabecular bone was thick and scattered with osteoblasts, and BMP2 and RUNX2 were positively expressed (Figure 5).

### 3.11 MicroCT

**FIGURE 6:**
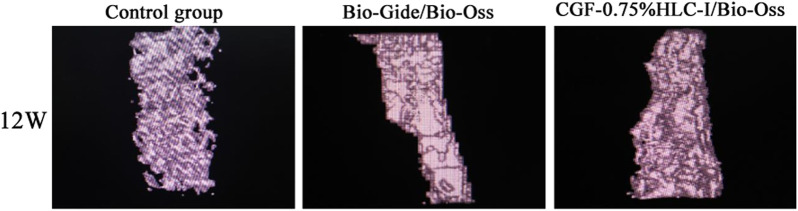
MicroCT images of control group, Bio-Gide/Bio-Oss group and CGF-0.75%HLC-I/Bio-Oss group (12 W).

The 12 W rabbit mandibles were scanned by MicroCT, and the osteocyte analysis parameters of the critical bone defect area of the rabbit mandibles in each group were obtained. The results showed that the BV/TV, BS/BV, Tb.Th, Tb.N of the Bio-Gide/Bio-Oss group and the CGF-0.75%HLC-I/Bio-Oss group were significantly than the control group. There was an increasing trend (*p* < 0.05). While Tb. Sp showed a decreasing trend (*p* < 0.05). However, there was no significant difference in BV/TV, BS/BV, Tb.Th, Tb.N, Tb. Sp between Bio-Gide/Bio-Oss group and CGF-0.75% HLC-I/Bio-Oss group (*p* < 0.05) (Table 3)., 3D representation of the constructed trabecular bone: Bio-Gide/Bio-Oss group and CGF-0.75%HLC-I/BioOss had more densely arranged trabecular bone compared to the control group (Figure 6).

**TABLE 3:** The cytotoxicity of CGF membrane and CGF-0.75%HLC-I composite biofilm was determined by CCK-8 (‾X ± S).

Material	24 h	48 h	72 h
CGF	74.75 ± 2.27	78.78 ± 1.78	93.20 ± 1.80
CGF-0.75%HLC-I	77.16 ± 1.08	82.34 ± 1.92	96.35 ± 1.68

### 3.12 The results of proteomic analysis of new bone tissue

**FIGURE 7:**
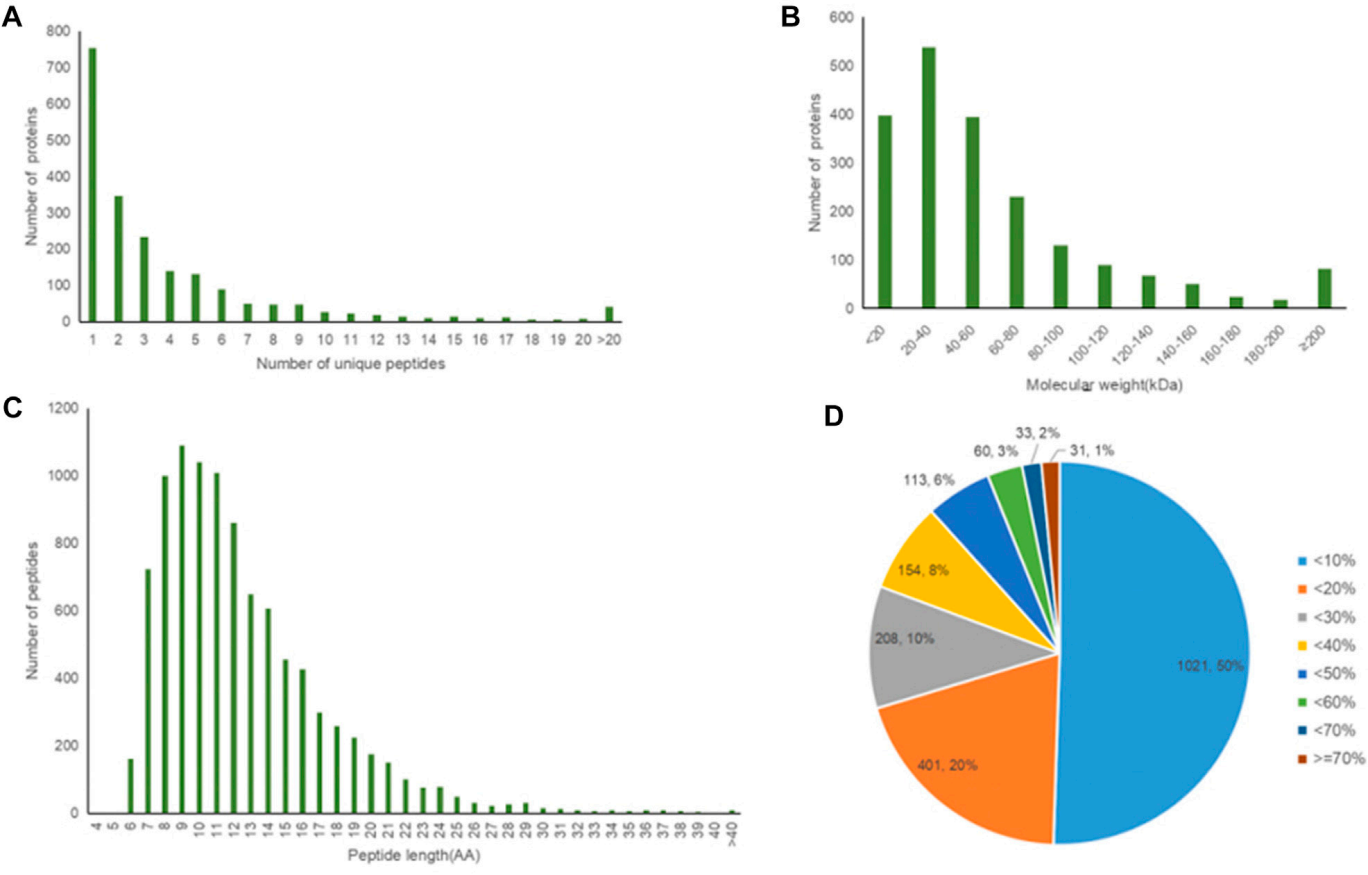
**(A–D)** Protein identification/Quantification overview.

**FIGURE 8:**
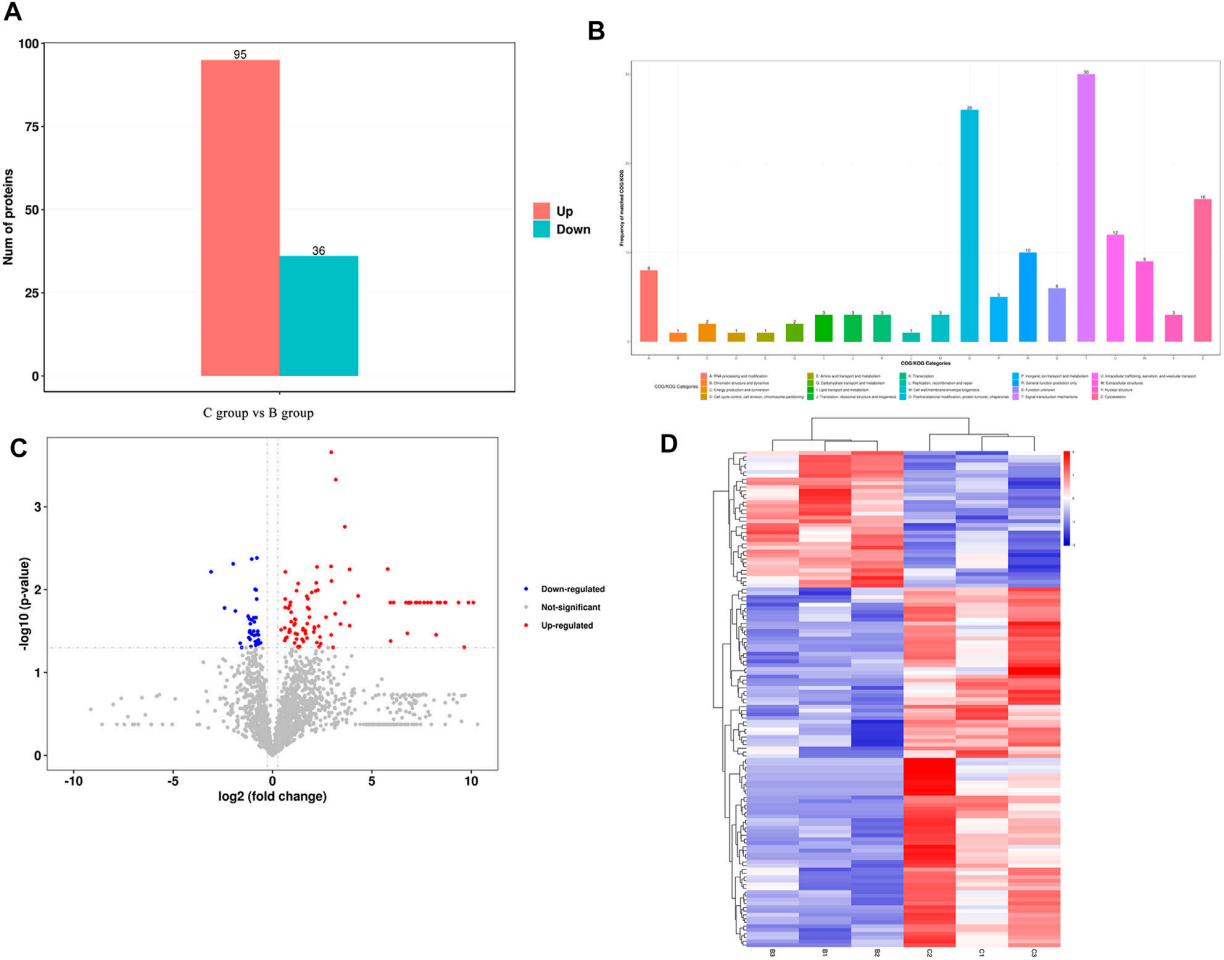
**(A)** Differentially expressed proteins between group **(C,B)**; **(B)** Number of proteins corresponding to the first 20 items of COG/KOG analys COG/KOG; **(C)** Volcano diagram of the DEPs between group **(C,B)**; **(D)** Clustering heat map of DEPs between group **(C,B)**.

**FIGURE 9:**
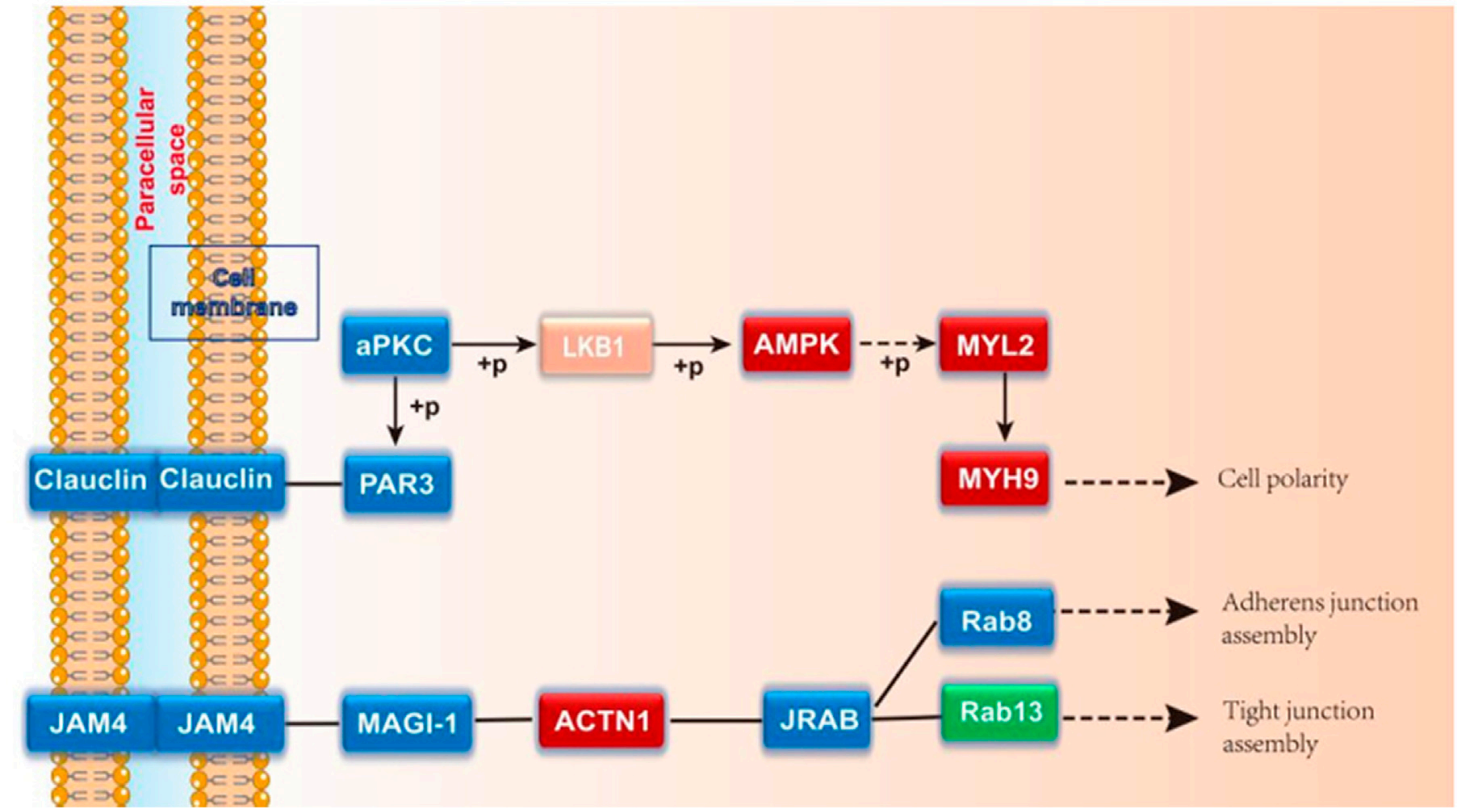
Schematic diagram of TJs pathway.

A total of 2021 proteins (groups) and 9601 polypeptides were identified. The distribution of protein and peptide identification scores, protein molecular, peptide length, and protein identification peptide coverage were shown in Figures 7A–D, respectively. The protein profile information was searched and matched in protein databases. There were 131 DEPs in the B and C groups, containing 95 up-regulated proteins and 36 down-regulated proteins (Figure 8A). Figure 8C showed the volcano plot of DEPs in group C compared to group B. Figure 8D showed the heat map of DEPs in the ratio of group C and group. Statistical analysis selected 8 DEPs with statistical significance, including 3 up-regulated proteins: ACTN1 (FC = 1.87, *p* = 0.014), BASP1 (FC = 1.56, *p* = 0.016), MYH9 (FC = 1.53, *p* = 0.041) and 5 down-regulated proteins: SPARC (FC = 0.58, *p* = 0.013), CALM (FC = 0.44, *p* = 0.038), KIF12 (FC = 0.52, *p* = 0.022), CDH13 (FC = 0.55, *p* = 0.010), EP841L2 (FC = 0.56, *p* = 0.046) (Table 4). Kyoto Encyclopedia of Genes and Genomes (KEGG) analysis database enrichment analysis results: ACTN1 and MYH9 are the main potential differential proteins related to osteogenesis, and they are enriched in the TJs pathway. In the TJs pathway, ACTN1 binds downstream to JRAB, which in turn binds to RAB13 and RAB8. Upstream of MYH9 are AMPK, p-AMPK and MYL2, AMPK indirectly phosphorylates MYL2, and MYL2 activates MYH9 (Figure 9).

**TABLE 4:** Differential expressed protein.

Gen name	Accession	Mean C	Mean B	*p*-Value	Fold Change
MYH9	G1SL68	118117390.8	77105026.51	0.04122,310,246	1.531902602
APARC	G1T2N1	6810491869	11832654100	0.01299013579	0.5755675618
ACTN1	A0A5F9DRU0	60145983.17	32109879.05	0.01421695244	1.873130169
CALM	P62160	1113160529	2545767146	0.03789906742	0.4372593661
BASP1	A0A5F9CFU9	95038895.1	61094898.26	0.01643238649	1.555594621
KIF12	G1TSY8	125602510.8	241713097.6	0.02178307525	0.5196346912
CDH13	G1TL92	52894520.29	97108904.65	0.009888347915	0.5446927908
EPB41L2	A0A5F9CHR4	54794547.05	98589173.11	0.04671912928	0.5557866581

### 3.13 CGF-0.75%HLC-Ⅰ-mediated tight junction pathway affects mandibular repair and regeneration in rabbits

#### 3.13.1 The results of immunohistochemical

**FIGURE 10:**
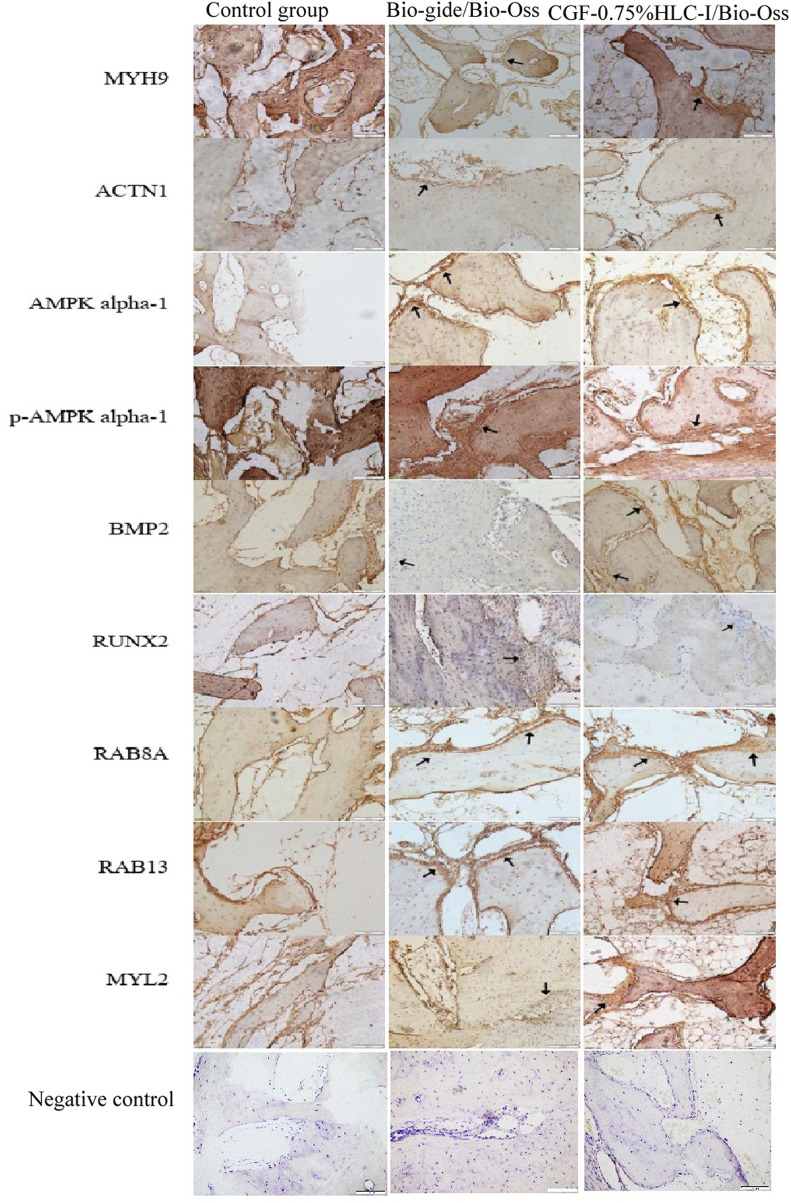
The immunohistochemical staining of tissues (arrow shows osteoblasts) (SP, ×200).

Immunohistochemical analysis of the positive expression of MYH9, ATCN1, BMP2, AMPK, p-AMPK, RUNX2, RAB8A, RAB13 and MYL2 in new bone tissue in groups A, B, and C, respectively. Osteoblasts can be clearly seen in the newly formed bone tissue in groups B and C. The expression level of MYH9 in group C was higher than that in group B (*p* < 0.01). The expression level in group A was higher than that in group B (*p* < 0.05). The expression level of ACTN1 in group A was higher than group B (*p* < 0.01). The expression level in group C was higher than that in group A and B (*p* < 0.001). The expression level of RAB8A in group B was higher than group A (*p* < 0.01). The expression levels of AMPK, p-AMPK, RUNX2, BMP2, RAB13 and MYL2 in the three groups were not statistically significant (Figure 10).

#### 3.13.2 The results of Western blot

**FIGURE 11:**
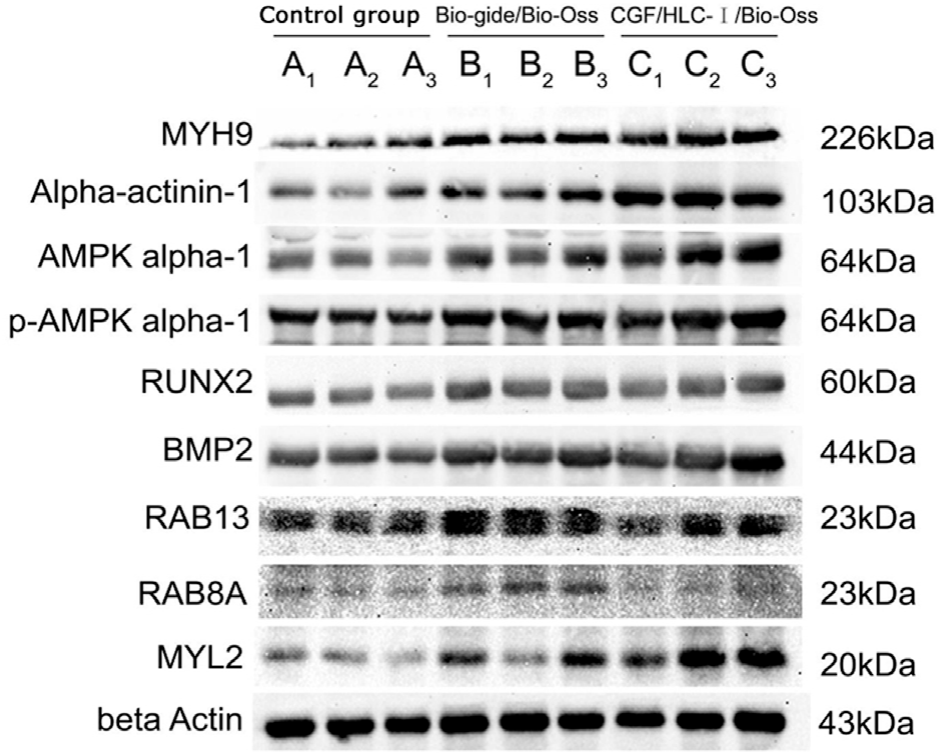
The Western blot analysis of osteoblasts.

The expression level of MYH9 and ACTN1 in group C was higher than group A (*p* < 0.01) and group B (*p* < 0.05). The expression level of AMPK and MYL2 in group C was higher than group A (*p* < 0.05). The expression levels of BMP2 in group C and group B were significantly higher than those in group A (*p* < 0.05). The expression levels of RAB13 in group B were significantly higher than those in group A (*p* < 0.01) and group C (*p* < 0.05). The expression levels of RUNX2, p-AMPK and RAB8A in the three groups were not statistically significant (Figure 11).

## 4 Discussion

Collagen is a kind of natural polymer material that can be used for bone defect repair and regeneration, and its biocompatibility is good. However, due to its high extraction cost, the research on it has only achieved a simplified bionic structure (Liao et al., 2021). Mingu K et al. found that collagen played a role in bone formation in the repair of calvarial defects in rabbits (Kim et al., 2021). Bio-Gide combined with Bio-Oss, a resorbable bone filler, can be used for bone regeneration and repair (Zhao et al., 2019). Bio-Gide has the function of promoting cell proliferation and has good biocompatibility (Schorn et al., 2019). Compared with the collagen membrane prepared from bovine collagen, its degradation degree was lower, which was more conducive to guiding bone regeneration (Neto et al., 2020). Bio-Gide combined with Bio-Oss was widely used in bone defect repair, such as implant bone regeneration, site preservation repair after tooth extraction (Zhang and Tu, 2021; Zhuang et al., 2021). Since Bio-Gide was expensive, it was necessary to find a material with good biocompatibility, strong osteoinductive ability and low cost. CGF has obvious advantages in the regeneration and repair of maxillofacial bone defects. At present, many researches combined CGF with other natural biomaterials, synthetic polymer materials, etc., in order to improve the performance of CGF.

HLC is a natural biological source of tissue engineering scaffold material. HLC become an ideal bioscaffold material for bone regeneration and repair with good cell biocompatibility, processability and low immunogenicity. However, its application was limited due to its low biological strength and uncontrollable biodegradation rate (Yu and Liu, 2019). It can be used in combination with other materials to improve the performance of the stent material. Shiyi H et al. used the freeze-dried sponge TGF-β3/HLC/CS prepared from HLC, transforming growth factor-β3 and chitosan to promote bone regeneration in rat calvarial defects (Huang et al., 2021). The HLC-BMP sponge prepared by Zhuoyue C (Chen et al., 2019) et al. can release growth factors, and it has been confirmed that it has an osteogenic induction effect in a rat calvarial defect repair model. The results of our previous study suggested that the combination of CGF and 0.75% HLC-I did not affect its good biocompatibility (Zhou et al., 2020). Consistent with the above findings, our study prepared different concentrations of CGF-HLC-I (0.1, 0.25, 0.5, 0.75, 1.00%) composite biomaterials by complex cross-linking CGF with different concentrations of HLC-I. MTT assay showed that the cell proliferation ability was the best when CGF was compounded with 0.75% HLC-I cross-linking. It was suggested that CGF-0.75%HLC-I composite biomaterial has the best biocompatibility with cells and has the ability to promote cell proliferation.

The new composite materials prepared by Hedong Y et al. can upregulate the expression of BMP2 to promote the growth of osteocytes and repair the mandibular defect in rabbits (Yu et al., 2018). RUNX2 is a type of transcription factor that can regulate bone development. Studies have found that RUNX2 can regulate a variety of osteocytes to participate in bone formation and bone remodeling, and can regulate the differentiation and proliferation of osteocytes (Orth et al., 2019; Sun et al., 2021; Wu et al., 2021). Ying P (Pang et al., 2021) et al. confirmed that activation of BMP2/RUNX2 signaling pathway can promote the proliferation and differentiation of human osteoblasts. In this study, the expression of BMP2 and RUNX2 protein were investigated by immunohistochemical in new bone tissue. In the Bio-Gide/Bio-Oss and CGF-0.75%HLC-I group, the positive expressions of BMP2 and RUNX2 increased at 4 W, 8 W, and 12 W after surgery. It was suggested that CGF-0.75%HLC-I has the effect of promoting osteogenesis.

Combined with other scholars at home and abroad, CGF may promote osteogenesis through the extracellular paracrine signaling channel through the growth factor TGF-β1 (Takahashi et al., 2019). In view of the above experiments, we further explored the repair mechanism of CGF-0.75%HLC-I -guided critical bone regeneration in rabbit mandible and compared and analyzed the repair mechanism with Bio-Gide. Teunissen M (Teunissen et al., 2018) et al. found 2,981 unique genes differentially expressed in the study of bone and cartilage regeneration, mentioning that the TJs signaling pathway was activated. However, there was no further evidence for a specific role of the TJs pathway in osteogenesis. The results of white matter analysis showed that 131 proteins with different expression levels were identified in CGF-0.75%HLC-Ⅰ/Bio-Oss group and Bio-Gide/Bio-Oss group including 95 up-regulated proteins and 36 down-regulated proteins. Three up-regulated proteins ACTN1, BASP1 and MYH9, and five down-regulated proteins SPARC, CALM, KIF12, CDH13 and EP841L2 were selected by Student’s t-test or chi-square test. It was suggested that the proteins regulated by CGF-0.75% HLC-I/Bio-Oss group and Bio-Gide/Bio-Oss group are different. Therefore, we speculated that the two metabolic pathways involved in mediating the regeneration and repair of critical bone tissue in the mandible of rabbits are also different.

There was a study found that ACTN1 was a potential risk of osteoporosis, and the main biological function of ACTN1 was to regulate the binding of actin cytoskeleton and calcium ions, so as to affect the ability of osteogenesis (Zeng et al., 2017). It can be seen that there was insufficient research evidence whether ACTN1 promoted osteogenesis or led to osteoporosis. However, our results showed that the expression of ACTN1 in CGF-0.75%HLC-Ⅰ/Bio-Oss group was higher than that in blank group and Bio-Gide/Bio-Oss group. It was speculated that ACTN1 can promote osteogenesis, that was, the high expression of ACTN1 can be used as a favorable proof that CGF-0.75%HLC-Ⅰ/Bio-Oss mediates the repair of bone tissue defects and bone tissue regeneration.

The enrichment analysis results of KEGG pathway suggested that there were four enrichment points in TJs pathway, in which the differential proteins ACTN1 and MYH9 are enriched in TJs pathway. AMPK and MYL2 are upstream proteins of MYH9. As an energy metabolism sensor, AMPK can regulate the differentiation of bone marrow mesenchymal stem cells. There was a study found that AMPK can phosphorylate RUNX to promote osteogenesis (Jiating et al., 2019). Yamamoto et al. (Yamamoto et al., 2014) once found that phosphorylated MYL2 was significantly induced into high expression in periodontal ligament cells during osteogenic differentiation. Inhibiting the expression of phosphorylated MYL2 can reverse the osteogenic differentiation of periodontal ligament cells. In this experiment, AMPK and MYL2 are upstream proteins of MYH9 in TJs pathway. The expression levels of AMPK and MYL2 are highest in CGF-0.75% HLC-I/Bio-Oss group. We speculated that AMPK can indirectly act on its downstream MYL2, phosphorylate it, and participate in the repair of bone tissue defects mediated by CGF-0.75% HLC-I. CGF-0.75%HLC-I induced the high expression of AMPK in the defective bone tissue, which in turn controls the polarity of osteoblasts and promotes the migration of osteoblasts and the transport of substances (Jiating et al., 2019). At the same time phosphorylation of MYL2, and finally achieve the role of promoting osteogenesis. Immunohistochemistry and Western blot analysis showed that MYH9, ACTN1, AMPK, p-AMPK, MYL2, RAB8A and RAB13 were expressed in each group. The expression levels of MYH9, ACTN1, AMPK and MYL2 were highest in the CGF-0.75%HLC-Ⅰ/Bio-Oss group. While RAB13 had the highest expression level in the Bio-Gide/Bio-Oss group. The high expression of MYL2 may activate its downstream MYH9, thereby promoting bone tissue regeneration. Based on the results of this experiment, RAB13 is highly expressed in Bio Gide/Bio-Oss group, while it is low expressed in other groups. The experimental results showed that MYH9 and ACTN1 were involved in different signal transduction processes in the TJs signaling pathway. AMPK, p-AMPK and MYL2 were upstream proteins of MYH9; the upstream protein of ACTN1 was MAGI-1, and the downstream proteins were JRAB, RAB8 and RAB13.

## 5 Conclusion

In conclusion, the mechanism of CGF-0.75%HLC-I/Bio-Oss repairing CSD was still different at the protein level compared with Bio-Gide/Bio-Oss. There were also differences in the metabolic pathways involved in the osteogenesis mechanism mediated by the two. In the TJs pathway, AMPK, MYL2 and MYH9 were up-regulated based on CGF-0.75%HLC-I, while AMPK activated MYL2 and MYH9. And by controlling cell polarity, promoting cell migration and material transport, and phosphorylating MYL2, it finally mediated the repair of rabbit mandibular CSD and promoted the mechanism of bone tissue generation.

Comparing the CGF-0.75% HLC-I/Bio-Oss group with the Bio-Gide/Bio-Oss group, a total of 8 classical protein differential expression distributions were excavated. Among them, ACTN1, BASP1, MYH9 were up-regulated; and SPARC, CALM, KIF12, CDH13 and EP841L were down-regulated. The results of differential protein combined with KEGG pathway enrichment showed that the main potential differential proteins related to osteogenesis were ACTN1 and MYH9, and both were enriched in the TJs pathway. CGF-0.75% HLC-I regulates the expression levels of AMPK, MYL2, MYH9 and ACTN1 through the TJs pathway, thereby promoting the repair of mandibular CSD. The results suggested that CGF-0.75%HLC-I/Bio-Oss materials can promote new bone formation, providing new ideas for the application of bone tissue engineering scaffold materials in oral clinics.

## Data Availability

The original contributions presented in the study are included in the article, further inquiries can be directed to the corresponding authors.

## References

[B1] Akcan S. K. Ünsal B. (2020). Gingival recession treatment with concentrated growth factor membrane: A comparative clinical trial. J. Appl. Oral Sci. 28, e20190236. 10.1590/1678-7757-2019-0236 32236353 PMC7105285

[B2] Arıcan G. Özmeriç A. Fırat A. Kaymaz F. Ocak M. Çelik H. H. (2022). Micro-ct findings of concentrated growth factors (cgf) on bone healing in masquelet's technique-an experimental study in rabbits. Arch. Orthop. Trauma Surg. 142, 83–90. 10.1007/s00402-020-03596-z 32945957

[B3] Chen Z. Zhang Z. Ma X. Duan Z. Hui J. Zhu C. (2019). Newly designed human-like collagen to maximize sensitive release of BMP-2 for remarkable repairing of bone defects. Biomolecules 9, 450. 10.3390/biom9090450 PMC6769454 31487971

[B4] Du C. Cui F. Z. Zhang W. Feng Q. L. Zhu X. D. de Groot K. (2000). Formation of calcium phosphate/collagen composites through mineralization of collagen matrix. J. Biomed. Mat. Res. 50, 518–527. 10.1002/(sici)1097-4636(20000615)50:4<518:aid-jbm7>3.0.co;2-w 10756310

[B5] Gao Y. Chen X. Tian T. Zhang T. Gao S. Zhang X. (2022). A lysosome-activated tetrahedral Nanobox for encapsulated siRNA Delivery. Adv. Mat., e2201731. 10.1002/adma.202201731 35511782

[B6] Hempel U. Möller S. Noack C. Hintze V. Scharnweber D. Schnabelrauch M. (2012). Sulfated hyaluronan/collagen I matrices enhance the osteogenic differentiation of human mesenchymal stromal cells *in vitro* even in the absence of dexamethasone. Acta Biomater. 8, 4064–4072. 10.1016/j.actbio.2012.06.039 22771456

[B7] Hong Y. J. Chun J. S. Lee W. K. (2011). Association of collagen with calcium phosphate promoted osteogenic responses of osteoblast-like MG63 cells. Colloids Surfaces B Biointerfaces 83, 245–253. 10.1016/j.colsurfb.2010.11.028 21177080

[B8] Hu K. Cui F. Lv Q. Ma J. Feng Q. Xu L. (2008). Preparation of fibroin/recombinant human-like collagen scaffold to promote fibroblasts compatibility. J. Biomed. Mat. Res. A 84, 483–490. 10.1002/jbm.a.31440 17618493

[B9] Huang S. Yu F. Cheng Y. Li Y. Chen Y. Tang J. (2021). Transforming growth factor-β3/recombinant human-like collagen/chitosan freeze-dried sponge primed with human periodontal ligament stem cells promotes bone regeneration in calvarial defect rats. Front. Pharmacol. 12, 678322. 10.3389/fphar.2021.678322 33967817 PMC8103166

[B10] Jiating L. Buyun J. Yinchang Z. (2019). Role of metformin on osteoblast differentiation in type 2 diabetes. Biomed. Res. Int. 2019, 1–6. 10.1155/2019/9203934 PMC6899291 31886264

[B11] Kim M. G. Lee J. H. Kim G. C. Hwang D. S. Kim C. H. Kim B. J. (2021). The effect of autogenous tooth bone graft material without organic matter and type I collagen treatment on bone regeneration. Maxillofac. Plast. Reconstr. Surg. 43, 17. 10.1186/s40902-021-00302-w 34143329 PMC8212298

[B12] Li W. Wang F. Dong F. Zhang Z. Song P. Chen H. (2021). CGF membrane promotes periodontal tissue regeneration mediated by hUCMSCs through upregulating TAZ and osteogenic differentiation genes. Stem Cells Int. 2021, 1–14. 10.1155/2021/6644366 PMC8360720 34394357

[B13] Li X. Yang H. Zhang Z. Yan Z. Lv H. Zhang Y. (2019). Concentrated growth factor exudate enhances the proliferation of human periodontal ligament cells in the presence of TNF-α. Mol. Med. Rep. 19, 943–950. 10.3892/mmr.2018.9714 30535499 PMC6323209

[B14] Liao J. Tian T. Shi S. Xie X. Ma Q. Li G. (2017). The fabrication of biomimetic biphasic CAN-PAC hydrogel with a seamless interfacial layer applied in osteochondral defect repair. Bone Res. 5, 17018. 10.1038/boneres.2017.18 28698817 PMC5496380

[B15] Liao X. Wang F.-k. Wang G.-l. (2021). Advances and challenges in bone tissue engineering scaffolds. Chin. Tissue Eng. Res. 25, 8.

[B16] Masuki H. Okudera T. Watanebe T. Suzuki M. Nishiyama K. Okudera H. (2016). Growth factor and pro-inflammatory cytokine contents in platelet-rich plasma (PRP), plasma rich in growth factors (PRGF), advanced platelet-rich fibrin (A-PRF), and concentrated growth factors (CGF). Int. J. Implant Dent. 2, 19. 10.1186/s40729-016-0052-4 27747711 PMC5005757

[B17] Mijiritsky E. Assaf H. D. Peleg O. Shacham M. Cerroni L. Mangani L. (2021). Use of PRP, PRF and CGF in periodontal regeneration and facial rejuvenation-A narrative review. Biol. (Basel) 2021, 317. 10.3390/biology10040317 PMC8070566 33920204

[B18] Neto A. M. D. Sartoretto S. C. Duarte I. M. Resende R. F. B. Neves Novellino Alves A. T. Mourão C. (2020). *In vivo* comparative evaluation of biocompatibility and biodegradation of bovine and porcine collagen membranes. Membr. (Basel) 10, 423. 10.3390/membranes10120423 PMC7765348 33333940

[B19] Orth M. Shenar A. K. Scheuer C. Braun B. J. Herath S. C. Holstein J. H. (2019). VEGF-loaded mineral-coated microparticles improve bone repair and are associated with increased expression of epo and RUNX-2 in murine non-unions. J. Orthop. Res. 37, 821–831. 10.1002/jor.24267 30835895

[B20] Pang Y. Liu L. Mu H. Priya Veeraraghavan V. (2021). Nobiletin promotes osteogenic differentiation of human osteoblastic cell line (MG-63) through activating the BMP-2/RUNX-2 signaling pathway. Saudi J. Biol. Sci. 28, 4916–4920. 10.1016/j.sjbs.2021.06.070 34466066 PMC8381068

[B21] Rochira A. Damiano F. Marsigliante S. Gnoni G. V. Siculella L. (2013). 3, 5-Diiodo-l-thyronine induces SREBP-1 proteolytic cleavage block and apoptosis in human hepatoma (Hepg2) cells. Biochimica Biophysica Acta - Mol. Cell Biol. Lipids 1831, 1679–1689. 10.1016/j.bbalip.2013.08.003 23948263

[B22] Sahin I. O. Gokmenoglu C. Kara C. (2018). Effect of concentrated growth factor on osteoblast cell response. J. Stomatol. Oral Maxillofac. Surg. 119, 477–481. 10.1016/j.jormas.2018.06.001 29885910

[B23] Sanders D. W. Bhandari M. Guyatt G. Heels-Ansdell D. Schemitsch E. H. Swiontkowski M. (2014). Critical-sized defect in the tibia: Is it critical? Results from the SPRINT trial. J. Orthop. Trauma 28, 632–635. 10.1097/bot.0000000000000194 25233157

[B24] Schorn L. Handschel J. Lommen J. Fp V. O. N. B. Depprich R. Kübler N. (2019). Evaluation of biocompatibility of different membrane surfaces using unrestricted somatic stem cells. Vivo 33 5, 1447–1454. 10.21873/invivo.11623 PMC6755023 31471391

[B25] Sun W. Li M. Xie L. Mai Z. Zhang Y. Luo L. (2021). Exploring the mechanism of total flavonoids of drynariae rhizoma to improve large bone defects by network pharmacology and experimental assessment. Front. Pharmacol. 12, 603734. 10.3389/fphar.2021.603734 34149403 PMC8210422

[B26] Takahashi A. Tsujino T. Yamaguchi S. Isobe K. Watanabe T. Kitamura Y. (2019). Distribution of platelets, transforming growth factor-β1, platelet-derived growth factor-BB, vascular endothelial growth factor and matrix metalloprotease-9 in advanced platelet-rich fibrin and concentrated growth factor matrices. J. Investig. Clin. Dent. 10, e12458. 10.1111/jicd.12458 31461225

[B27] Teunissen M. Riemers F. M. van Leenen D. Groot Koerkamp M. J. A. Meij B. P. Alblas J. (2018). Growth plate expression profiling: Large and small breed dogs provide new insights in endochondral bone formation. J. Orthop. Res. 36 1, 138–148. 10.1002/jor.23647 28681971 PMC5873274

[B28] Wang Y. Li Y. Gao S. Yu X. Chen Y. Lin Y. (2022). Tetrahedral framework nucleic acids can alleviate taurocholate-induced severe acute pancreatitis and its subsequent multiorgan injury in mice. Nano Lett. 22, 1759–1768. 10.1021/acs.nanolett.1c05003 35138113

[B29] Wei Z. Huang X. S. Chen Z. (2020). Application and research progress of concentrated growth factor in oral clinic. Int. J. Stomatology 47, 9.

[B30] Wu Y. Han J. Wen S. (2021). Mechanism of action of Runx2 gene during fracture healing. Chin. Tissue Eng. Res. 25, 6.

[B31] Yamamoto T. Ugawa Y. Yamashiro K. Shimoe M. Tomikawa K. Hongo S. (2014). Osteogenic differentiation regulated by Rho-kinase in periodontal ligament cells. Differentiation 88 (2-3), 33–41. 10.1016/j.diff.2014.09.002 25278479

[B32] Yu H. Chen Y. Mao M. Liu D. Ai J. Leng W. (2018). PEEK-biphasic bioceramic composites promote mandibular defect repair and upregulate BMP-2 expression in rabbits. Mol. Med. Rep. 17, 8221–8227. 10.3892/mmr.2018.8867 29658566 PMC5983999

[B33] Yu L. Liu X. (2019). Progress in the application of humanoid collagen in bone tissue regeneration and repair. J. Xinxiang Med. Coll. 3, 4.

[B34] Zeng Y. Zhang L. Zhu W. He H. Sheng H. Tian Q. (2017). Network based subcellular proteomics in monocyte membrane revealed novel candidate genes involved in osteoporosis. Osteoporos. Int. 28 10, 3033–3042. 10.1007/s00198-017-4146-5 28741036 PMC5812280

[B35] Zhang T. Tian T. Lin Y. (2021). Functionalizing framework nucleic-acid-based nanostructures for biomedical application. Adv. Mat., e2107820. 10.1002/adma.202107820 34787933

[B36] Zhang Z. Tu B. (2021). Research on bio-OSS Collagen combined with Bio-Guide biofilm for site preservation after anterior tooth extraction. Chin. health Stand. Manag. 12, 3. 10.3969/j.issn.1674-9316.2021.18.016

[B37] Zhao L. P. Hu W. J. Xu T. Zhan Y. L. Wei Y. P. Zhen M. (2019). Two procedures for ridge preservation of molar extraction sites affected by severe bone defect due to advanced periodontitis. Beijing Da Xue Xue Bao Yi Xue Ban. 51, 579–585. 10.19723/j.issn.1671-167X.2019.03.030 31209434 PMC7439019

[B38] Zhou M. Chen X. Qiu Y. Chen H. Liu Y. Hou Y. (2020). Study of tissue engineered vascularised oral mucosa-like structures based on ACVM-0.25% HLC-I scaffold *in vitro* and *in vivo* . Artif. Cells Nanomed. Biotechnol. 48, 1167–1177. 10.1080/21691401.2020.1817055 32924619

[B39] Zhuang G. Mao J. Yang G. Wang H. (2021). Influence of different incision designs on bone increment of guided bone regeneration (Bio-Gide collagen membrane +Bio-OSS bone powder) during the same period of maxillary anterior tooth implantation. Bioengineered 12 1, 2155–2163. 10.1080/21655979.2021.1932209 34057023 PMC8806879

